# Jump performance and handgrip strength do not reflect acute fatigue in elite trail runners after the 2023 world trail running championship

**DOI:** 10.3389/fspor.2025.1506067

**Published:** 2025-06-13

**Authors:** Diego Jaén-Carrillo, Felipe García-Pinillos, Pedro E. Alcaraz, Cristian Marín-Pagán, Francisco J. Martínez-Noguera, Peter A. Federolf

**Affiliations:** ^1^ Department of Sport Science, University of Innsbruck, Innsbruck, Austria; ^2^ Department of Physical Education and Sport, University of Granada, Granada, Spain; ^3^ Sport and Health University Research Center (iMUDS), Granada, Spain; ^4^ Department of Physical Education, Sport and Recreation. Universidad de La Frontera, Temuco, Chile; ^5^ Research Center for High Performance Sport, Catholic University of Murcia (UCAM), Murcia, Spain

**Keywords:** endurance, performance analysis, stiffness, testing, trail running, biomechanics, mountain running, off-road

## Abstract

**Purpose:**

Fatigue manifests as a decline in maximal voluntary contraction (MVC), driven by both central and peripheral factors. Studies have shown reduced maximum isometric force in knee extensor and plantar flexor muscles after ultradistance trail running. This study aimed to assess the effectiveness of jump tests [countermovement jump [CMJ] and 30 cm-height drop jump [DJ]] and handgrip strength tests in detecting acute neuromuscular fatigue among elite marathon trail runners following the 2023 Trail Running World Championship.

**Methods:**

Eight elite athletes (4 males and 4 females; height: 168 ± 8.62 cm; weight: 56.18 ± 9.28 kg; age: 32.98 ± 5.43 years) were recruited. Paired samples T-Test analyses are used.

**Results:**

No significant differences were found in all jumping variables except for the Reactive Strength Index (RSI) (t = 2.44, df = 7, *p* = 0.045), with a large effect size (ES = 0.862). Handgrip strength test analyses showed no significant reductions post-run.

**Conclusion:**

RSI decreased while other performance variables remained unchanged. This suggests the athletes’ fatigue had less impact on their performance in these tests than anticipated, showing a less reactive jump strategy (*p* < 0.05 for RSI) after the competition.

## Introduction

Trail running, defined as a competitive event primarily in natural environments with less than 20% paved paths, spans diverse landscapes like mountains and deserts. Distances range from a few kilometres to over 80 km, without elevation limits (1). Of note, the 2023 Trail World Championship involved a 45.2 km course with 3132 m elevation gain (2). Over the last two decades, the sport has surged by 2,394%, with a 231% increase in the last decade (3) and its official recognition by World Athletics in 2015 (4) has significantly contributed to its growth and professionalization. This acknowledgment has sparked discussions about the possibility of trail running being included as an Olympic sport in the foreseeable future, underscoring its bright and promising trajectory.

Fatigue is characterized by a decrease in maximal voluntary contraction (MVC), with both central and peripheral origins. Bainbridge in 1931 (5) first differentiated these origins, noting that central fatigue stems from the central nervous system's impaired ability to activate motor units. This impairment may involve reduced motor unit recruitment or a drop in their discharge frequency to below the tetanic fusion rate. Research indicates significant central fatigue during prolonged exercises such as running for over two hours, with a noticeable reduction in voluntary activation percentage (6). Neuromuscular fatigue occurs when the central nervous system or muscle tissues cannot generate expected force during exercise (7). Several studies have observed reductions in maximum isometric force production in knee extensor and plantar flexor muscles following ultradistance trail running (8–14) Similarly, Espeit and colleagues also documented central fatigue after trail runs ranging from 40 to 170 km (15). The decline in muscle force is attributed to impaired maximal voluntary activation, decreased sarcolemma excitability, disruptions in excitation-contraction coupling, and cross-bridge alterations. Central mechanisms, especially at the supraspinal level, are particularly influential in the observed strength reduction following ultra-endurance running (Millet et al., 2018). Factors such as hypoglycaemia, diminished catecholamine levels, increased core body temperature, accumulation of cerebral ammonia, and alterations in brain neurotransmitter levels can influence central fatigue. Additionally, feedback from contracting muscles can alter spinal interactions, impacting mechanisms like Ia disfacilitation and inhibition via Ib, III, and IV afferent fibres (Millet et al., 2018). Traditionally, the evaluation of fatigue using vertical jumps has been tested in laboratory settings (16). However, recent advancements have introduced user-friendly, low-cost portable equipment that allows fatigue evaluation during training sessions and race events, directly in the field (17, 18). The mobile application *MyJump* is widely used for researchers and practitioners given its validity and reliability data in assessing jumping variables (18–23) and vertical stiffness (24).

During running legs function like springs, compressing and decompressing due to body mass (25, 26). This action facilitates the storage of mechanical energy during the eccentric phase of the stance and its subsequent release as elastic energy in the concentric phase. Central to this mechanism of elastic energy utilization are the concepts of lower-limb stiffness (27) and the stretch-shortening cycle (SSC) (28) highlighting the crucial neuromuscular elements involved. In evaluating lower-limb power, stiffness, and reactive strength, exercises such as the countermovement jump (CMJ) and drop jump (DJ) serve as effective, straightforward tools. These tasks engage athletes in slow and rapid SSC actions, respectively, shedding light on their neuromuscular capabilities (25). A comprehensive assessment of the DJ, for instance, considers both the jump height and the ground contact time, key for absorbing and generating force. Here, the reactive strength index (RSI), defined as the ratio of flight time to ground contact time, emerges as a pivotal metric, offering insights into an athlete's ability to swiftly transition from eccentric to concentric contractions and to produce maximal force in minimal time (29). The analysis of vertical jumps extends beyond performance metrics to include indicators of fatigue (29–32). These assessments play a vital role in gauging lower body reactive strength, evaluating lower-limb stiffness, and monitoring neuromuscular fatigue (33). Previous studies indicate that endurance-trained runners exhibit an improvement in countermovement jump (CMJ) performance following a fatiguing running test. This enhancement in CMJ is linked to increases in peak power and a lesser reduction in eccentric maximum strength (34). Such enhancements are thought to result from the simultaneous occurrence of post-activation potentiation (PAP) and muscular fatigue. PAP refers to a temporary improvement in muscle performance following its contraction history (35). Moreover, the response to PAP following running appears to be specific to endurance-trained athletes (34, 36). Of note, Del Rosso and colleagues evaluated CMJ height during a self-paced 30 km trial, evaluating the CMJ each 5 km performance of endurance-trained runners (37). While a reduction in speed existed during the 30 km run, an increase from the baseline and maintenance towards the end of the trial was reported in jump performance, being attributed to a potentiation effect (37). However, the evaluation of jump tests to assess fatigue in elite trail runners after a world championship has never been performed.

The handgrip strength test has been also used in previous studies for monitoring fatigue. In this regard, the decreased muscular force in those muscles not involved in the exercise reveals supraspinal fatigue (6, 38). Central fatigue induced by exercise is manifested by a decrease in muscle activation (39). The maintenance of handgrip strength, even showing a trend to increase indicates the importance of central mechanisms in maintaining a certain level of force (40). It would be expected that central fatigue occurred after exhausted running protocols. However, previous research found no differences in handgrip strength before and after a running-induced fatigue protocol (41). Then, why endurance runners may not show an impairment in handgrip strength or jumping tests? observed a decrease in muscle stiffness alongside enhanced responsiveness to electrical stimulation. They suggested that these phenomena reflect concurrent mechanisms involving the activation of fast-twitch fibres and the fatigue of slow-twitch fibres during marathon trail running (42). In a more recent study, Márquez and colleagues argued that changes in CMJ performance may not effectively measure acute fatigue in endurance-trained runners (43). It is established that endurance training not only increases phosphorylation of myosin light chains in slow fibres but also enhances fatigue resistance (44). Trail runners, who typically undergo long-duration, low-intensity training, might not be accurately assessed for fatigue using conventional methods like CMJ and DJ vertical jump tests, along with handgrip strength evaluations. Current research on assessing acute neuromuscular fatigue in elite trail runners post-world championship using vertical jumps and handgrip tests remains limited.

The aim of the current study was to assess applicability of common tests for acute neuromuscular fatigue with elite marathon trail runners in a situation that was certainly fatiguing, specifically, after competing in the 2023 Trail Running World Championship. Considering the above information, we hypothesized that CMJ and DJ height, particularly power output and RSI would show substantial changes immediately after completing the championship run compared to measurements in non-fatigued state one day earlier. In addition, a handgrip strength test was included as a measure of central fatigue; but here we expected smaller effects.

## Methods

### Participants

The eight athletes (3 Spanish and 1 German males, and 4 Spanish females: 168 ± 8.62 cm; weight: 56.18 ± 9.28 kg; age: 32.98 ± 5.43 years; Trail Running experience: 7.40 ± 8 2.95 years; weekly volume in the season: 92.00 ± 17.52 km) were recruited through convenience and snowball non-probability sampling methods. Eligibility for participation in the study required athletes to be older than 18 years old and competitors in the Trail Running World Championship 2023, and to have no musculoskeletal injuries. Athletes who withdrew before finishing the full distance were exempt from post-race measurements and consequently were excluded from further analysis (dropout *n* = 3). All of them volunteered to participate and provided written consent after receiving detailed verbal and written explanations of the study protocol and understanding the associated risks. The Host University ethics committee approved the protocol (Certificate 36/2023), adhering to the Declaration of Helsinki's principles.

One previous study on fatigue after an ultramarathon reported an effect size of d = 1.4 for the reduction of squat jump height (45). Effect sizes in the range d ≥ 1.3 imply that a sample size of 6 athletes would suffice to detect a fatigue. This was calculated with G-Power® (46) setting the alpha-threshold to 0.05 and requiring a power of 80% (one-tailed calculation). The current study used 8 athletes, which implies that effect sizes of d > 0.98 can be detected at a power of 80%.

### Experimental design

**Figure 1:**
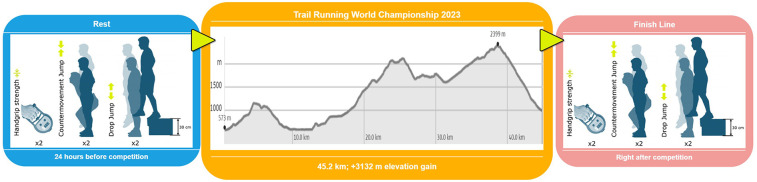
Timeline of the testing protocol.

A quasi-experimental design was employed to evaluate the impact of a 45-km trail running world championship on neuromuscular fatigue. The athletes underwent an identical testing protocol: (i) 24 h prior to the competition (Short Trail at the 2023 Mountain and Trail Running World Championships, Innsbruck, Austria), they were to perform two trials of maximal handgrip strength, CMJ, and DJ from 30 cm height (DJ30); then (ii) they were required to complete the full competition, and (iii) to repeat the handgrip strength test and the jumping tests as outlined in (i) immediately after they cross the finish line (time after finish: 4.88 ± 1.36 min) (Figure 1). The best performance was taken for further analysis. Considering the importance of this competition (World Championship), all athletes followed their normal routine of training and pre-competitive rest, not interfering with their planning. The day before the competition the athletes reported that they performed activation runs at low intensity and carbohydrate-rich diet.

### Measurements

#### CMJ and DJ as indicators of lower-limb neuromuscular fatigue

Before the jump tests, athletes completed a warmup comprised of 5 min pedalling on a bike ergometer at a comfortable intensity followed by dynamic stretching, body-weight squats and pogo jumps. After, athletes undertook three submaximal familiarization trials of both the CMJ and DJ exercises. Each jump was performed with hands on hips and using a self-selected depth for the countermovement (22, 47). After familiarization, participants executed three maximal efforts for both CMJs and DJs. For the CMJs, they were instructed to achieve maximum height following a quick countermovement. Here, jump height (in cm), power (in W) and force (in N) were considered for further analyses. For the DJs, participants stepped off a 30-cm box and then aimed to jump as high as possible while minimizing ground contact time, as outlined previously (48), and jump height, RSI, and stiffness were considered. Each test included a 2-minute period of passive recovery while trials were separated by 30 s (49). Each jump was captured with an iPhone 13 (Apple Inc., CA, USA) at a frame rate of 240 Hz and subsequently analysed according to the guidelines provided by MyJump3. Although the app offers the possibility of identifying the key events of the jumps (i.e., ground contacts and landings) automatically, all the events were identified manually by the same researcher. The iPhone was mounted on a tripod using a smartphone clamp (MCPIXI Universal, Manfrotto, Italy) and positioned 1.5 m in front of the athlete (18).

#### Supraspinal fatigue—handgrip strength test

The handgrip strength assessment was conducted in accordance with the guidelines reported in (50) and the optimal grip setting was also determined (51). Handgrip strength measurements (in kilograms) were captured using a digital hand dynamometer (TKK 5101 Grip D; Takey, Tokyo, Japan). Athletes were motivated to exert their maximum handgrip strength.

### Statistical analyses

Data analysis was conducted using the Jamovi software package (version 2.3.26, The Jamovi Project). The Shapiro–Wilk test was employed to assess the normality of the variables, all of which showed a normal distribution. Subsequently, the t-test for related samples was used to determine the differences in jump variables for CMJ and DJ before and after the competition, with a significance threshold of *p* < 0.05. Also, Student's t-test for independent samples was carried out for all the variables to identify differences in performance between sexes. When the equal of variances was violated, Mann–Whitney *U* test was used. Additionally, effect sizes were reported and interpreted according to Cohen's guidelines (52); specifically, an effect size of 0.2 was considered small, 0.5 medium, and ≥0.8 large.

## Results

### CMJ and DJ as indicators of lower-limb neuromuscular fatigue

**Figure 2:**
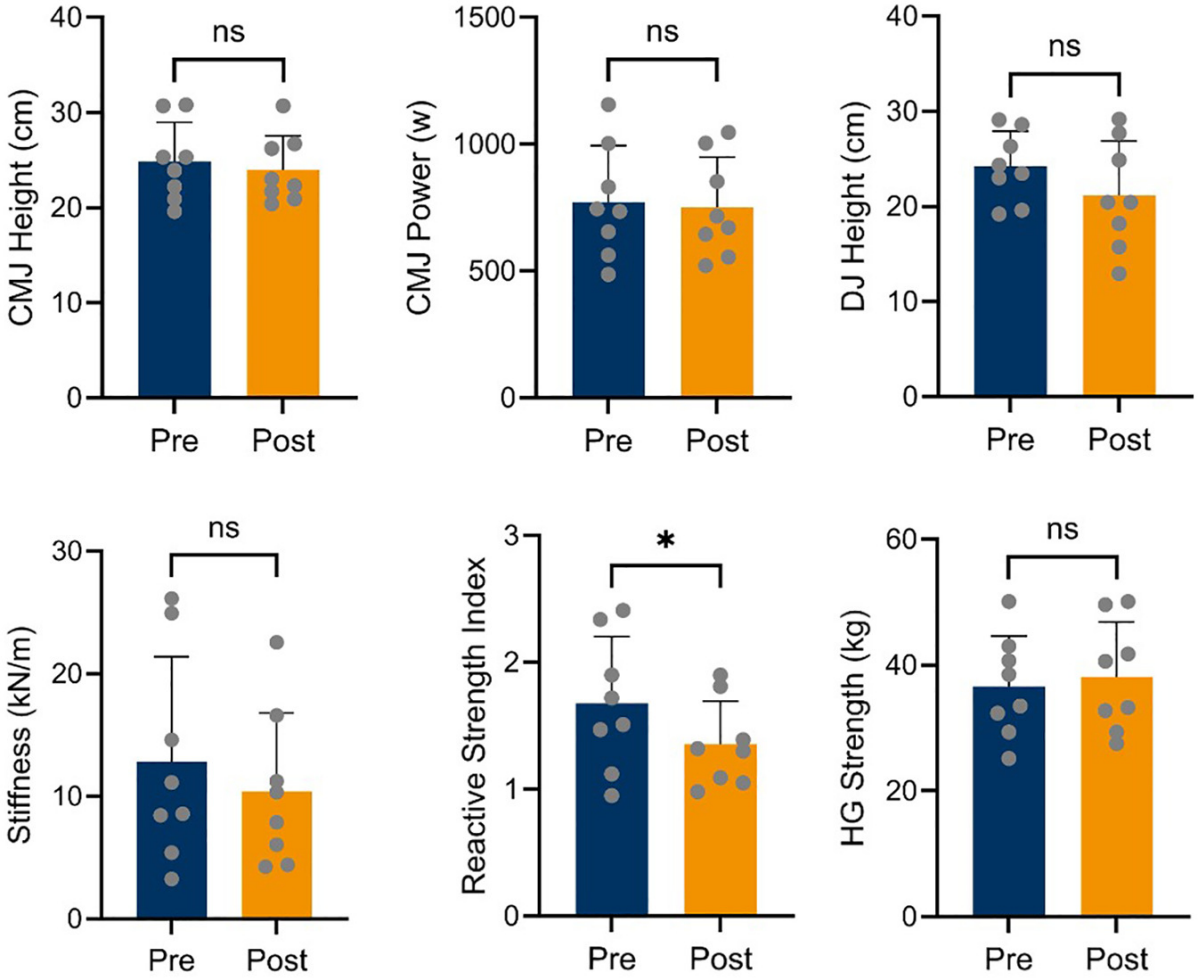
Jumping variables values before (pre) and after the race (post) derived from countermovement jump (CMJ) and drop jump (DJ) and handgrip (HG) strength test. RSI stands for reactive strength index. *Denotes *p* < 0.05, ns denotes non significant.

**Figure 3:**
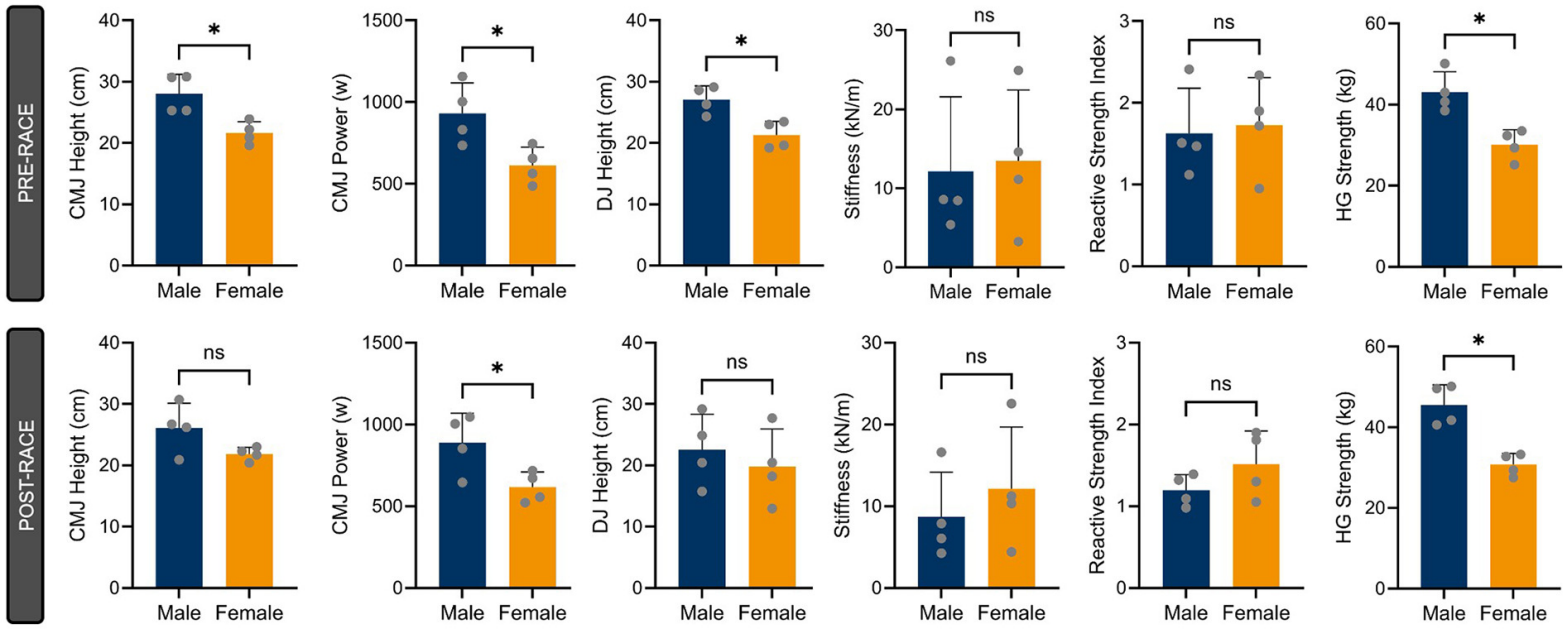
Jumping variables values PRE-RACE and POST-RACE derived from countermovement jump (CMJ) and drop jump (DJ) and handgrip (HG) strength test. RSI, reactive strength index. *Denotes *p* < 0.05, ns denotes non significant.

Figure 2 shows the values of all the variables assessed. Paired samples *T*-Test analyses revealed no significant differences for all variables analysed but for RSI (t = 2.44, df = 7, *p* = 0.045), also reporting a large effect size (ES = 0.862). When analysing differences between sexes, significant differences were found for power output (i.e., pre and post-race) for the CMJ (t = −2.66, df = 6, *p* = 0.037) showing males a reduction from pre-race (932.05 ± 186.24 w) to post-race testing (887.52 ± 181.51 w), while females slightly increase from pre to post-race testing (612.52 ± 112.18 w and 616.38 ± 92.72 w, respectively), reporting a very large effect size (ES = −1.881). Although significant differences were also found between males and females for CMJ and DJ height in the pre-race measurements, no significant differences were found after the race due to a reduction in height in males for both jumps, while female performance remained unchanged (Figure 3).

### Handgrip strength test as indicator of supraspinal fatigue

The values for handgrip strength before and after competition are plotted in Figure 2. Paired samples *T*-test analysis revealed no differences before and after the competition. When analysing differences between sexes (Figure 3), significant differences were found for handgrip strength (U = 0.00, df = 6, *p* = 0.029) showing males an increase from pre-race (Median = 41.90 kg, IQR = 4.62 kg) to post-race testing (Median = 45.70 kg, IQR = 8.23 kg), while females remain unchanged from pre to post-race testing (Median = 30.90 kg, IQR = 4.32 kg and Median = 31.10 kg, IQR = 4.00 kg, respectively), reporting a very strong effect [Rank-biserial Correlation (r_rb) = 1.000].

## Discussion

The purpose of this study was to assess fatigue in elite trail runners following the 2023 Mountain and Trail Running World Championships. The researchers used jump tests and leg stiffness measurements to evaluate peripheral fatigue, and a handgrip strength test to assess central fatigue. Consistent with previous studies (41), handgrip strength was not significantly reduced after the run. Contrary to our initial hypothesis, the fatigue effects observed in the jump tests were smaller than anticipated and were non-significant for all variables except RSI. This not only suggests that the type of fatigue experienced by the athletes had less impact on their performance in these tests than initially expected, but also a change in jump strategy when fatigued given that they were less reactive (*p* < 0.05 for RSI) after the competition, that might be originated due to a deterioration in the stretch-shortening cycle and, therefore, an indication of the inability of elite trail runners to generate explosive force efficiently after such competition. These findings align with a recent study indicating that acute changes in jump performance may not be a suitable measure for evaluating acute fatigue in endurance-trained runners (43), a conclusion that may also apply to elite marathon trail runners.

Several topics arising from these results warrant further discussion: first, a likely prerequisite for this observation is the high level of conditioning of these athletes and their adeptness at managing fatigue. Then, it is astonishing that in jump tests, which substantially involve the same muscle groups as needed in trail running, no larger declines were observed, apart from RSI. However, when analysed by sex, males generated significantly lower power after competition, while power values for females remained unchanged. Although the findings, when analysing the sample as a whole, suggests that neuromuscular mechanisms might play a role in the apparent fatigue resistance of these muscles (43), differences in jump performance between sexes aligned with previous findings, where it was concluded that females exhibited less peripheral fatigue in the plantar flexors than males did after a 110 km ultra-trail-running race and males demonstrated a greater decrease in maximal force loss in the knee extensors (13).

### Conditioning towards adeptness at managing fatigue

Regulatory mechanisms in the brain control athlete performance to prevent catastrophic physiological failures (53, 54). These pre-emptive pacing strategies are influenced by environmental conditions, physiological status, fuel levels, previous experience, affect, cognitive capacity, and the presence of competitors (55, 56). These strategies likely include a reserve capacity, allowing athletes to increase power output in the race's final stages. The ability to increase pace at the end of a race depends on conserving energy and managing body temperature, staying close to competitors to challenge them at the finish. Long-distance runners, for example, aim to maintain an intensity that conserves fuel for the race's end, possibly replenishing anaerobic capacity or maintaining oxygen utilization at a level that allows for a final push or unexpected need for increased power (57). If unable to maintain this strategy due to a fast competitor pace, athletes must decide whether to increase effort, risking physiological strain, or slow down to avoid collapse, as recent evidence indicates most do (57).

Specific trail running training over years may play a significant role at managing fatigue during the most competitive events in elite trail runners, what may explain the lack of a significant fatigue after the 2023 Trail World Championship found here. The significant reduction in RSI post-race, despite unchanged jump height or force measures, suggests that elite trail runners adjust their movement strategy under fatigue, possibly by unconsciously adopting a more controlled landing technique. This aligns with previous findings showing that RSI is a more sensitive marker of fatigue than absolute jump performance (29, 32). Of note, RSI is also associated with metabolic cost in long-distance runners, particularly in older athletes, suggesting that its role extends beyond fatigue assessment to running efficiency (31). Future research should examine how long-term trail running adaptations enhance fatigue resistance, pacing strategies, and the ability to maintain RSI across different race distances and competition levels.

### Specificity of muscle fatigue

Years of trail running training may have led to neuromuscular adaptations that contribute to the ability of performing explosive strength tasks even when fatigued from a competitive run (58). Recent investigations into neuromuscular fatigue during trail running emphasize central (59) and particularly supraspinal (15) contributions. The Flush Model, originally suggested for ultra marathon running (60) builds on the concept of a central governor model (61, 62). It suggests that during the progression of fatigue, the central nervous system establishes a security reserve (63) to prevent physiological harm. This internal pacing strategy (64) reserves pools of motor units that remain available for emergencies. It is likely that neuromuscular adaptations in highly conditioned athletes optimize motor unit recruitment, predominantly relying on type I fibres for endurance tasks, while preserving type II fibres for explosive actions. This could explain why RSI was significantly reduced post-race in elite trail runners despite no change in CMJ or DJ height, indicating that neuromuscular fatigue affected landing strategy rather than overall power output. The findings contrast with those of Tanneau et al. (31), where RSI was primarily linked to running efficiency, particularly in older athletes, rather than acute fatigue. These differences suggest that RSI may serve distinct roles depending on the athlete population and context: as a marker of fatigue in high-intensity trail running and as an indicator of efficiency in endurance-trained master athletes.

Such task specificity in the recruitment of motor units could explain why fatigued muscles can still perform certain movement tasks at a near-normal level, a phenomenon observed in previous research (65). A previous study also showed that endurance runners are able to maintain, and even improve, CMJ performance (i.e., height and power production) during and after a 30 km run (37). The authors attributed this enhancement in jump performance to a PAP effect (37). It seems that the higher the level of the athlete, the shorter time they need to benefit from PAP effects, between 5 and 7 min (66). Jump performance was assessed the day before the competition and right after the completion of the marathon trail running world championship (time after finish: 4.88 ± 1.36 min). Considering the short time elapsed between the end of the race and the measurement of jump performance, the lack of difference in jump performance might be attributed to PAP effects. The high level of task specificity in neural recruitment is likely a characteristic of extensive task-specific training, potentially distinguishing elite trail runners from other endurance athletes. Moreover, whether these adaptations are unique to elite-level conditioning or also present in lower-level trail runners remains an open question for future studies.

### Limitations and future research

This study provides valuable insights into the fatigue mechanisms of elite trail runners, yet several limitations must be considered when interpreting the results. Firstly, the sample was limited to elite-level athletes who participated in the 2023 Mountain and Trail Running World Championships, which restricts the generalizability of the findings to lower-level or recreational trail runners. The relatively small sample size, although representative of this highly specialized population, may also limit statistical power and hinder the detection of subtle sex-related differences in fatigue responses. Additionally, the study primarily utilized jump tests and handgrip strength to assess neuromuscular fatigue. While these measures are informative, they may not comprehensively capture all aspects of fatigue in trail running, especially those related to metabolic, proprioceptive, or central fatigue factors. It is also well known that an increase of 1°C environmental and muscle temperature enhances short duration neuromuscular performance (i.e., vertical jump) from 2% to 5% (67). Although this might also be a potential explanation for the results here reported, this was not taken into account, remaining such explanation unknown. Another limitation is the lack of consideration of individual differences in training backgrounds, biomechanics, or race strategies, all of which could influence fatigue responses and performance. Future research should aim to address these limitations by expanding the sample to include amateur and sub-elite trail runners, allowing for a broader understanding of fatigue mechanisms across different performance levels. Specifically, investigating sex differences in fatigue resistance, especially in lower-limb neuromuscular function (i.e., RSI), is essential, as previous research suggests that females may exhibit different fatigue responses compared to males. Furthermore, exploring fatigue mechanisms across various trail race distances would provide insight into how race duration and terrain type influence fatigue and performance. Incorporating additional fatigue markers, such as muscle stiffness measurements or metabolic indicators, alongside neuromuscular assessments like RSI, would offer a more comprehensive understanding of fatigue in endurance athletes. Additionally, examining how RSI and other neuromuscular markers respond to trail-specific fatigue factors, such as prolonged eccentric loading, varying terrain, and proprioceptive demands, could help uncover the mechanisms behind performance maintenance despite fatigue. Investigating long-term adaptations in SSC mechanics following repeated exposure to high-intensity trail running, and exploring potential recovery interventions to mitigate SSC impairments, would further contribute to optimizing training and recovery strategies for trail runners. Ultimately, these research efforts will improve our understanding of fatigue mechanisms in trail running and may inform more effective approaches to performance enhancement and injury prevention in this challenging sport.

## Conclusions

This study investigated fatigue in elite trail runners following the Short Trail event at the 2023 Mountain and Trail Running World Championships by using jump and handgrip strength tests. While handgrip strength remained unchanged, RSI significantly decreased (*p* < 0.05), suggesting a deterioration in the stretch-shortening cycle and impaired neuromuscular efficiency. Differences in fatigue responses between sexes align with previous research, highlighting reduced peripheral fatigue in female athletes. These findings support the notion that trail running conditioning plays a critical role in fatigue management and warrants further investigation into the underlying neuromuscular mechanisms and long-term adaptations.

## Data Availability

The datasets presented in this study can be found in online repositories. The names of the repository/repositories and accession number(s) can be found in the article/Supplementary Material.
